# A 3′ UTR polymorphism g.1618 G > A in the *MAFA* gene modulates miR-3678-3p binding and enhances meat production in sheep via the MAFA/GHR/JAK2 pathway

**DOI:** 10.1186/s12711-025-01024-7

**Published:** 2025-12-22

**Authors:** Cuiyu Lai, Dandan Tan, Xuewen Han, Yu Fu, Jinlin Chen, Xiaofan Yang, Xuesong Shan, Yang Chen, Huaizhi Jiang

**Affiliations:** College of Animal Science and Technology, Jilin Agricultural University, Changchun, 130118 China

## Abstract

**Background:**

MAFA (musculoaponeurotic fibrosarcoma oncogene Family A) is a specific transcriptional activator of the insulin gene (*INS*), playing a critical role in regulating insulin secretion and thereby indirectly influencing growth and development in animals. Previously, MAFA’s role in meat production remained speculative, despite its known function in endocrine regulation. However, our recent publication provides direct evidence linking MAFA to muscular phenotypes in sheep. These findings support a direct role for MAFA in muscle growth, complementing its canonical role in insulin-mediated growth regulation. To further investigate this proposition, we conducted genotype–phenotype association analyses to assess the potential impact of *MAFA* polymorphisms on meat production performance in sheep. In parallel, we employed a combination of dual-luciferase reporter assays, transcriptome profiling, and ChIP-PCR to dissect the underlying molecular mechanisms. These findings were further validated in ovine myoblasts.

**Results:**

We found that the g.1618 G/A polymorphism in the 3' UTR of the *MAFA* gene is associated with meat production in sheep. Individuals with the GG genotype exhibited a 36.9% higher proportion of *longissimus dorsi* muscle mass compared to those with the AA genotype. Further analysis, including binding site prediction and dual-luciferase reporter assays, revealed that this mutation may regulate *MAFA* translation efficiency by altering the binding affinity of miR-3678-3p. Subsequently, ChIP-PCR experiments confirmed that the growth hormone receptor (*GHR*) gene is a direct target of the transcription factor MAFA. By conducting miR-3678-3p transfection and *MAFA* overexpression experiments in sheep myoblasts, we further validated the miR-3678-3p/*MAFA*/*GHR* regulatory axis and the classical GHR/JNK signaling pathway. These findings elucidate the molecular mechanism by which the g.1618 G/A polymorphism in the *MAFA* gene affects meat production in sheep, providing a novel molecular marker with potential application in molecular breeding for improved meat performance in sheep.

**Conclusions:**

In summary, The G/A polymorphism at position g.1618 in the 3′ UTR of the *MAFA* gene affects the binding of miR-3678-3p, thereby regulating the expression of the transcription factor MAFA at the post-transcriptional level. MAFA, in turn, directly influences the transcription of its target gene *GHR*, which affects JAK2 phosphorylation, ultimately regulating myoblast proliferation and muscle growth.

**Supplementary Information:**

The online version contains supplementary material available at 10.1186/s12711-025-01024-7.

## Background

As a vital component of global meat production, lambs hold a prominent place in the culinary traditions of many regions due to its distinctive flavor and high nutritional value [1]. Improving meat yield and quality remains a primary objective in sheep breeding, with significant implications for the sustainability and efficiency of livestock production [2]. With advances in molecular biology theory and technology, the elucidation of genetic mechanisms underlying productive traits has laid a solid foundation for improving livestock performance. This progress has enabled the application of molecular breeding strategies, such as marker-assisted selection (MAS) and genome wide association studies (GWAS), which overcome the limitations of conventional phenotype-based selection, including low efficiency and long generation intervals. These approaches facilitate early selection and precise improvement of genetic resources, thereby markedly accelerating the breeding process [3, 4]. Skeletal muscle mass cannot be measured non-invasively in vivo, and traditional breeding programs must rely on post-mortem evaluation. This approach not only precludes individual selection but also limits selection intensity due to slaughter capacity constraints, ultimately slowing genetic gain. As a result, MAS has become an essential complement to classical selection strategies. Although numerous functional genes have been associated with muscle growth and meat traits, relatively few markers have been successfully translated into practical tools for livestock breeding [5, 6]. In sheep, genetic polymorphisms in growth hormone (*GH*), insulin-like growth factor 1 (*IGF1*), and myostatin (*MSTN*) have been extensively studied and linked to growth and meat production traits [7–10].

MAFA (musculoaponeurotic fibrosarcoma oncogene Family A), a member of the MAF transcription factor family, was initially identified in pancreatic β-cells, where it binds to the *INS* gene promoter and enhances *INS* transcription [11, 12]. Given insulin's pivotal role in metabolism, MAFA may indirectly influence animal growth and development through modulation of insulin secretion [13]. Intriguingly, *MAFA* is also more abundantly expressed in skeletal muscle compared with other tissues [14, 15], suggesting an additional, more direct role in muscle growth beyond its canonical endocrine functions. Indeed, our recent study provided the first direct evidence that MAFA contributes to muscle production traits in sheep. MAFA expression in skeletal muscle positively correlates with muscle mass, and integrative ChIP-Seq and RNA-Seq analysis revealed that MAFA binds to regulatory regions of genes involved in calcium signaling and receptor tyrosine kinase pathways—including INSR (insulin receptor) and EGFR (epidermal growth factor receptor)—thus implicating it in muscle growth regulation [15].

To further elucidate the regulatory mechanisms of MAFA in muscle growth, we conducted genotype–phenotype association analyses and identified a single nucleotide polymorphism (SNP), g.1618 G > A, in the 3′ untranslated region (3′ UTR) of *MAFA*, which is significantly associated with carcass traits in sheep. Individuals carrying the GG genotype exhibited a 36.9% higher proportion of *longissimus dorsi* muscle mass compared to those with the AA genotype. Functional assays including dual-luciferase reporter assay and miRNA binding site analysis indicated that this SNP may modulate MAFA translation by altering the binding efficiency of miR-3678-3p.

In downstream mechanism studies, although our previous work identified EGFR and INSR as potential downstream targets, MAFA binding occurred only within intronic regions—suggesting a limited role in direct transcriptional control. In contrast, leveraging ChIP-Seq data from our prior publication along with ChIP-PCR validation in this study [15], we identified *GHR*—a gene widely recognized for its key role in mediating animal growth—as a direct transcriptional target of MAFA, with MAFA binding to its promoter region.

Subsequent experiments involving miR-3678-3p mimic transfection and *MAFA* overexpression in ovine myoblasts validated the regulatory axis of miR-3678-3p/MAFA/GHR and its downstream signaling via the classical GHR/JNK pathway. These findings provide mechanistic insights into how the g.1618 G/A polymorphism in MAFA affects meat production in sheep and offer a novel molecular marker with potential utility in precision breeding for improved carcass performance.

## Methods

### Animals and phenotypic measurements

A total of 230 six-month-old F_1_ crossbred lambs (Australian White × Small-tailed Han sheep), weighing between 40 and 56 kg, were randomly selected. All animals were housed under identical conditions and fed according to the nutritional guidelines of the National Research Council (NRC, 2007). After overnight fasting, live weight was recorded prior to slaughter. Carcass weight and blood samples were collected post-slaughter. *Longissimus dorsi* muscle from left carcass was excised and weighed. Intramuscular fat (IMF), meat color, shear force, pH, and cooking loss were determined as described by Han et al. [16]. Muscle samples were either fixed in 4% paraformaldehyde, snap-frozen in liquid nitrogen, or used fresh for subsequent analyses.

### Serum insulin quantification

Serum was obtained by centrifuging 1 mL of anticoagulated whole blood at 3000–4000 rpm for 10–15 min. Insulin levels were measured using a commercial ELISA kit (0420A23, Ji-inhi, China) according to the manufacturer’s instructions. Absorbance was read at 450 nm using a multifunctional microplate reader (CLARIOstar Plus, BMG LABTECH, Germany).

### DNA extraction and genotyping

Genomic DNA was extracted from 25 mg of *longissimus dorsi* tissue using a tissue DNA extraction kit (D0063, Beyotime, China). DNA purity and concentration were assessed using a UV spectrophotometer. Fragment of the 3′ UTR from *MAFA* gene was amplified and sequenced by the Sanger method. Sequences for genomic DNA amplification are listed in Table S1.

### RNA-seq and bioinformatic analysis

Total RNA was extracted from *longissimus dorsi* of five GG and five AA homozygous animals. mRNA was isolated using magnetic beads, followed by first- and second-strand cDNA synthesis. Libraries were sequenced using the Illumina NovaSeq™ 6000 platform. Raw reads were filtered with fastp to generate high-quality clean data. HISAT2 was used for genome alignment, and gene annotation was performed through sequence comparison methods [17]. Transcript abundance was calculated as fragments per kilobase of transcript per million mapped reads (FPKM) using RSeQC (v4.0.0) [18]. DESeq2 was used with median-of-ratios normalization to account for differences in sequencing depth and RNA composition across samples [19]. Differentially expressed genes (DEGs) were identified using the following criteria: fold change (FC) ≥ 2 or ≤ 0.5, and adjusted *P* < 0.05 (Benjamini–Hochberg FDR).

### Dual-luciferase reporter assay

A 133 bp *MAFA* 3′ UTR fragment containing either the G or A allele at position g.1618 was synthesized (GenScript) and cloned into the Psicheck2.0 vector (C8021, Promega, USA). Plasmids (1 μg/μL) carrying either *MAFA*-G or *MAFA*-A, together with 40 pmol of miR-3678-3p mimic or negative control (NC), were co-transfected into HEK293T cells. After 48 h, luciferase activity was assessed using a dual-luciferase reporter assay kit (JKR23008-50 T, GeneCreate, China). Firefly luciferase activity was normalized to Renilla luciferase. Sequences for miR-3678-3p mimic and NC were:

mimic: CUGCAGAGUUUGUACGGACCGG/CCGGUCCGUACAAACUCUGCAG

NC: UCACAACCUCCUAGAAAGAGUAGA/UCUACUCUUUCUAGGAGGUUGUGA.

### Quantitative real-time PCR (qRT-PCR)

Total RNA was extracted from muscle using TRIzol reagent. Purity and integrity were verified spectrophotometrically. cDNA was synthesized and qRT-PCR was performed using SYBR Premix Ex Taq™ II on a CFX-96 real-time PCR system (Bio-Rad, USA). mRNA levels were calculated using the 2^-ΔΔCt method. Primer sequences are provided in Table S1.

### Western blotting

Total protein was extracted from tissues or cells and quantified. Proteins were denatured in loading buffer and separated by SDS-PAGE, then transferred to PVDF membranes. Membranes were blocked with 5% non-fat milk and incubated with primary antibodies at 4 °C overnight, followed by secondary antibody incubation at room temperature for 2 h. Signals were visualized using chemiluminescence and quantified with ImageJ (v1.54 g) [20]. Primary antibodies included: MAFA (bs-4279R), GHR (bs-11502R), JAK2 (bs-6092R), and p-JAK2 (bs-1832R) (all from Bioss, Beijing, China).

### Myoblast isolation, culture and differentiation

Fresh *longissimus dorsi* tissue was collected from sheep individuals homozygous for the AA genotype at the g.1618 G/A locus. One gram of tissue was washed in sterile saline containing 1% penicillin–streptomycin, minced, and digested with 1 mg/mL type I collagenase for 1 h. After centrifugation, cells were further digested with 0.25% trypsin for 15 min. Digestion was terminated with growth medium (DMEM/F12 + 10% FBS + antibiotics), and cells were filtered through 300- and 100-mesh sieves. Cells were cultured at 37 °C in 5% CO₂ incubator. When myoblasts reached 90% confluence, differentiation was initiated with DMEM/F12 and 2% horse serum. Myogenic differentiation was evaluated by calculating the fusion index—the proportion of nuclei in myosin-positive, multinucleated myotubes.

### Cell transfection

Myoblasts were cultured to confluence in 6-well plates and allowed to differentiate for three days [15]. Upon initiation of differentiation, cells were transfected using Lipofectamine 2000 (Invitrogen, Carlsbad, USA) with miR-3678-3p mimic/NC and pcDNA3.1-MAFA/empty vector (E0648, Sigma-Aldrich, USA). Cells were harvested 48 h post-transfection.

### EdU proliferation assay

Forty-eight hours post-transfection, cell proliferation was assessed using an EdU kit (SFClick™ EdU-488, Abcell, China). Cells were incubated with 50 μM EdU for 2 h, fixed with 4% paraformaldehyde, and permeabilized with 0.5% Triton X-100. EdU incorporation was visualized using Click-iT reaction and counterstained with Hoechst 33,342. Fluorescence microscopy was used for observation.

### Immunofluorescence

Cultured cells were fixed in 4% paraformaldehyde, then permeabilized with 0.2% Triton X‑100 (in PBS) for 10 min at room temperature. After blocking for 1.5 h at room temperature with 5% BSA in PBS, cells were incubated overnight at 4 °C with myosin primary antibody (bs‑10905R, Bioss, China). Following three PBS washes, samples were incubated at room temperature with Cy3‑conjugated secondary antibody (A0521, Beyotime, China). Nuclei were counterstained with DAPI (C1341S, Beyotime, China) for 10 min, and images were captured using an Olympus CK40 fluorescence microscope. Myogenic differentiation was assessed by calculating the fusion index (percentage of nuclei within myosin-positive multinucleated myotubes) [15].

### ChIP-qPCR

Approximately 100 mg of *longissimus dorsi* muscle tissue was sliced into sections less than 1 mm thick on ice. The samples were fixed in freshly prepared PBS containing 1% formaldehyde (F8775, Sigma-Aldrich, USA) for 5–15 min on ice. Cross-linking was quenched by adding 1/20 volume of 2.5 M glycine to a final concentration of 125 mM, followed by gentle agitation on ice for 5 min. Samples were washed three times with 1 × PBS and centrifuged at 1,000 × g for 5 min at 4 °C. The supernatant was thoroughly removed, and the pellet was used to prepare whole-cell lysates. Genomic DNA was fragmented by sonication to an appropriate size range. Immunoprecipitation was performed using an anti-MAFA antibody (#79,737, Cell Signaling Technology, USA), and IgG served as a negative control. Bound chromatin was eluted, reverse cross-linked, and the DNA was purified. Based on our previously obtained ChIP-seq data for *GHR* (SRA: PRJNA1135757) [15], primers targeting the *GHR* promoter region were designed. Quantitative PCR was conducted using the recovered DNA as a template. Enrichment was quantified using the 2^-ΔΔCt method, with ChIP signals normalized to input signals to calculate fold enrichment.

### Statistical analysis

Data are presented as mean ± SEM. For comparisons among multiple genotypes (GG, AA, and GA), group variances were tested using Levene’s test to verify homogeneity (all *P* > 0.05), and data were screened for outliers using the interquartile range (IQR) method. As both assumptions were met, we applied one-way ANOVA using the model: *Y*_*ij*_ = *μ* + *α*_*i*_ + *ε*_*ij*_, where *Y*_*ij*_ is the phenotype, μ represents the overall mean,* α*_*i*_ is the effect of genotype i, and *ε*_*ij*_*∼*N(0,σ^2^) denotes residual error. We calculated η^2^ as a measure of the proportion of total phenotypic variance accounted for by genotype (overall effect size in the ANOVA model). If the ANOVA yielded statistical significance, we conducted post hoc multiple comparisons using the Student–Newman–Keuls (SNK) test. For these pairwise comparisons, we quantified effect sizes as standardized mean differences (Cohen’s d) to reflect the magnitude of difference between two genotype groups.

All statistical analyses were performed using SPSS v27.0 [21], with *P* < 0.05 denoting statistical significance. Graphical outputs were generated with GraphPad Prism v9.5 [22].

## Results

### The g.1618 G/A polymorphism in the 3′ UTR of the MAFA gene is associated with longissimus dorsi muscle proportion

**Fig. 1:**
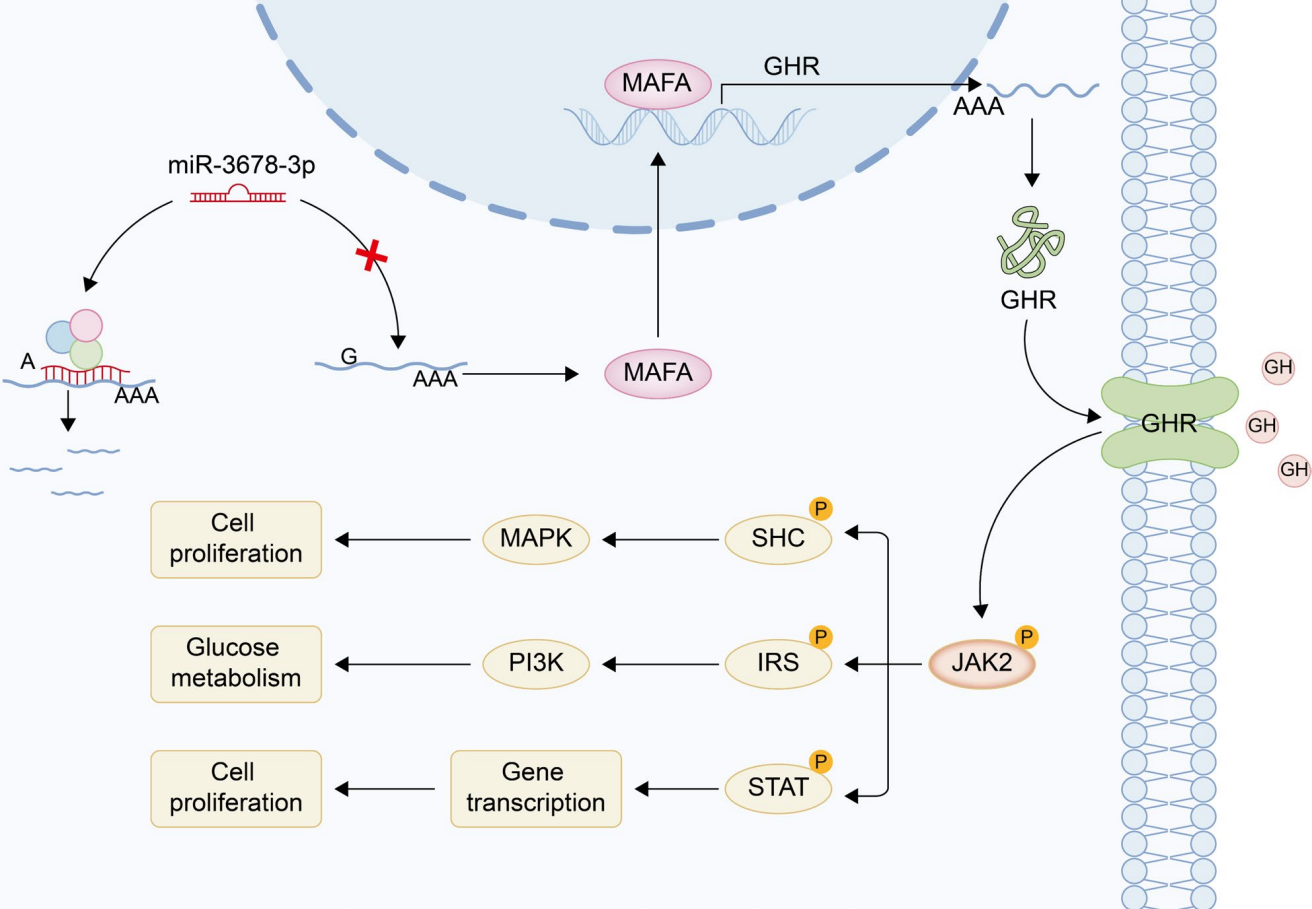
The g.1618 G/A polymorphism in the 3′UTR of the *MAFA* gene affects the proportion of *longissimus dorsi* muscle. **a** Schematic representation of the *MAFA* gene structure. **b** Sanger sequencing chromatograms showing genotypes at the g.1618 site of the *MAFA* gene. **c** Proportion of *longissimus dorsi* muscle in individuals with different genotypes (n = 230; GG = 72, AA = 48, GA = 110). **d** Serum insulin levels in individuals with different genotypes (n = 60; GG = 18, AA = 12, GA = 30). **e** Meat quality traits across different genotypes (n = 30; GG = 9, AA = 6, GA = 15). Data with error bars represent mean ± SEM. Statistical significance between the GG genotype and the genotype with the greatest difference is indicated with significance as follows: ns, not significant; *P < 0.05; **P < 0.01; ***P < 0.005; ****P < 0.001; *****P < 0.0005

According to the sheep reference genome (NC_056062.1), *MAFA* spans 2,699 bp on chromosome 9 (Chr9:14,181,994–14,184,692), contains a single exon without introns, and encodes a 351-amino acid protein (Fig. 1a). The 3′ UTR of *MAFA* harbors abundant polymorphic loci, and due to the absence of introns, nearly all genetic variation is localized within the 3′ UTR. Sanger sequencing was performed to genotype the g.1618 site in 230 lambs (Fig. 1b), followed by analysis of the association between genotype and *longissimus dorsi* muscle proportion. Individuals with the GG genotype exhibited a significantly higher muscle proportion than those with AA or GA genotypes (Table S2; *P* < 0.0001, Cohen’s *d* = 2.04 and 1.39, respectively), with a 36.9% increase compared to AA animals (Fig. 1c). The estimated allele substitution effect was α = 0.2005 [23].

Given that MAFA is a transcriptional activator of the *INS* gene, we initially hypothesized that this genotype-associated variation in muscle mass might be mediated by differences in insulin expression. To test this, serum insulin levels were measured in a subset of 60 animals. No significant differences were observed among genotypes (Fig. 1d), suggesting that the effect of the *MAFA* gene on skeletal muscle proportion is likely independent of insulin regulation.

To evaluate potential effects of the g.1618 G/A polymorphism on meat quality, we analyzed muscle samples from 30 individuals. Aside from a significantly higher pH at 24 h postmortem (pH_24_) in AA genotypes compared to GG animals (Table S2, *P* < 0.05, Cohen’s *d* = -0.30), no significant differences were found for meat color parameters (L*, a*, b*), shear force, cooking loss, IMF, or muscle fiber diameter (Fig. 1e).

### MiR-3678-3p binding at the g.1618 SNP modulates MAFA expression

**Fig. 2:**
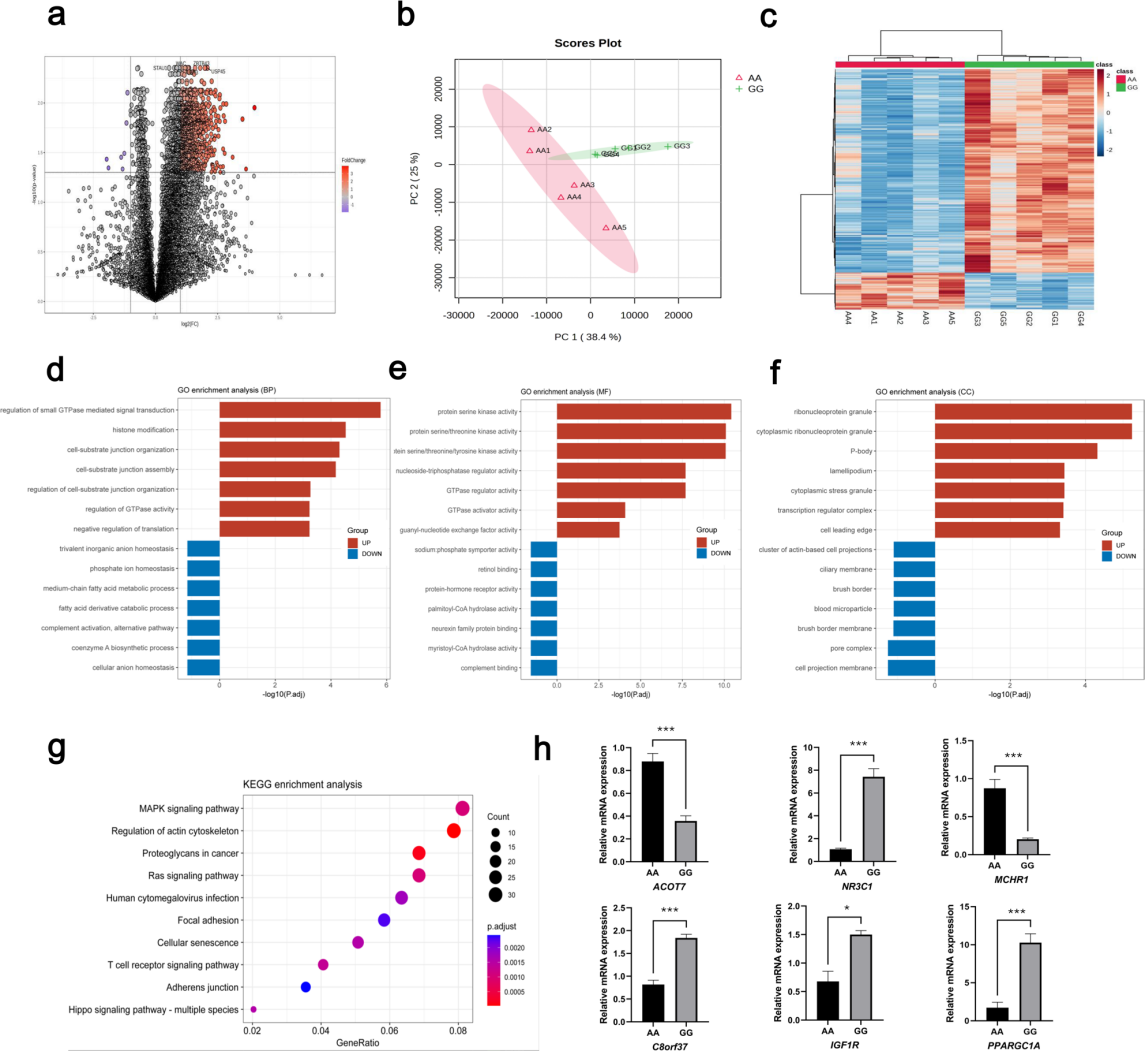
MiR-3678-3p regulates MAFA protein expression by binding to the g.1618 site in the 3′UTR of the *MAFA* gene. **a** Predicted pairing between miR-3678-3p and the *MAFA* 3′ UTR sequence containing either the A or G allele at position 182. **b** Dual-luciferase reporter assay evaluating the binding affinity of miR-3678-3p to *MAFA* 3′ UTR variants (n = 3). **c** Relative expression levels of miR-3678-3p in the *longissimus dorsi* muscle of individuals with different *MAFA* genotypes (n = 3). **d** Quantitative PCR analysis of *MAFA* mRNA expression in individuals with different genotypes (n = 3). **e** Western blot analysis of MAFA protein levels in individuals with different genotypes. Data are presented as mean ± SEM. Statistical significance is indicated as follows: ns, not significant; *P < 0.05; **P < 0.01; ***P < 0.005; ****P < 0.001; *****P < 0.0005

Given that mutations in the 3′ UTR can affect post-transcriptional regulation, we investigated the mechanistic role of the g.1618 G/A polymorphism in the *MAFA* 3′ UTR. Using our previously published miRNA-seq data from sheep muscle tissue (Gene Expression Omnibus accession number GSE113173) [24], we employed the miRDB prediction tool [25] to identify miRNAs with potential binding sites in the *MAFA* 3′ UTR. Notably, miR-3678-3p was predicted to bind at the region containing the g.1618 site (Fig. 2a), with the A allele creating a highly stable 8-mer seed match with the first nucleotide of the miRNA seed region [26]. This mutation is predicted to enhance the affinity of miR-3678-3p for the *MAFA* 3′ UTR, potentially suppressing *MAFA* translation.

To experimentally validate this interaction, we synthesized reporter constructs containing either the G or A allele at the g.1618 site and performed dual-luciferase assays. The construct harboring the G allele exhibited significantly higher luciferase activity compared to the A allele (*P* < 0.0005, Fig. 2b), consistent with increased repression by miR-3678-3p in the presence of the A allele.

We next quantified the expression levels of miR-3678-3p and MAFA mRNA in longissimus dorsi muscle from lambs with GG (n = 3) and AA (n = 3) genotypes. No significant differences in miR-3678-3p levels were observed between genotypes (Fig. 2c), and although *MAFA* mRNA showed a trend toward higher expression in GG individuals, the difference was not statistically significant (Fig. 2d). These results suggest that the g.1618 G/A polymorphism does not substantially affect *MAFA* transcription.

To determine whether the mutation impacts protein translation, we conducted Western blot analysis to quantify MAFA protein levels. MAFA protein abundance was significantly higher in GG individuals compared to AA animals (*P* < 0.001, Fig. 2e), indicating that the genotype-dependent differences in *MAFA* expression are mediated at the translational level.

Together, these results demonstrate that miR-3678-3p exhibits differential binding to the *MAFA* 3′ UTR depending on the g.1618 allele, modulating MAFA protein expression through translational regulation in sheep muscle tissue.

### Transcriptomic analysis of different genotypes

Based on our results showing that the MAFA gene does not affect skeletal muscle mass through insulin-mediated indirect effects, we propose that MAFA may instead play a direct regulatory role in muscle growth. To explore the downstream mechanisms following miR-3678-3p-mediated regulation of MAFA protein abundance, we performed transcriptomic profiling of the *longissimus dorsi* muscle from five GG and five AA genotype lambs using high-throughput RNA sequencing.

**Fig. 3:**
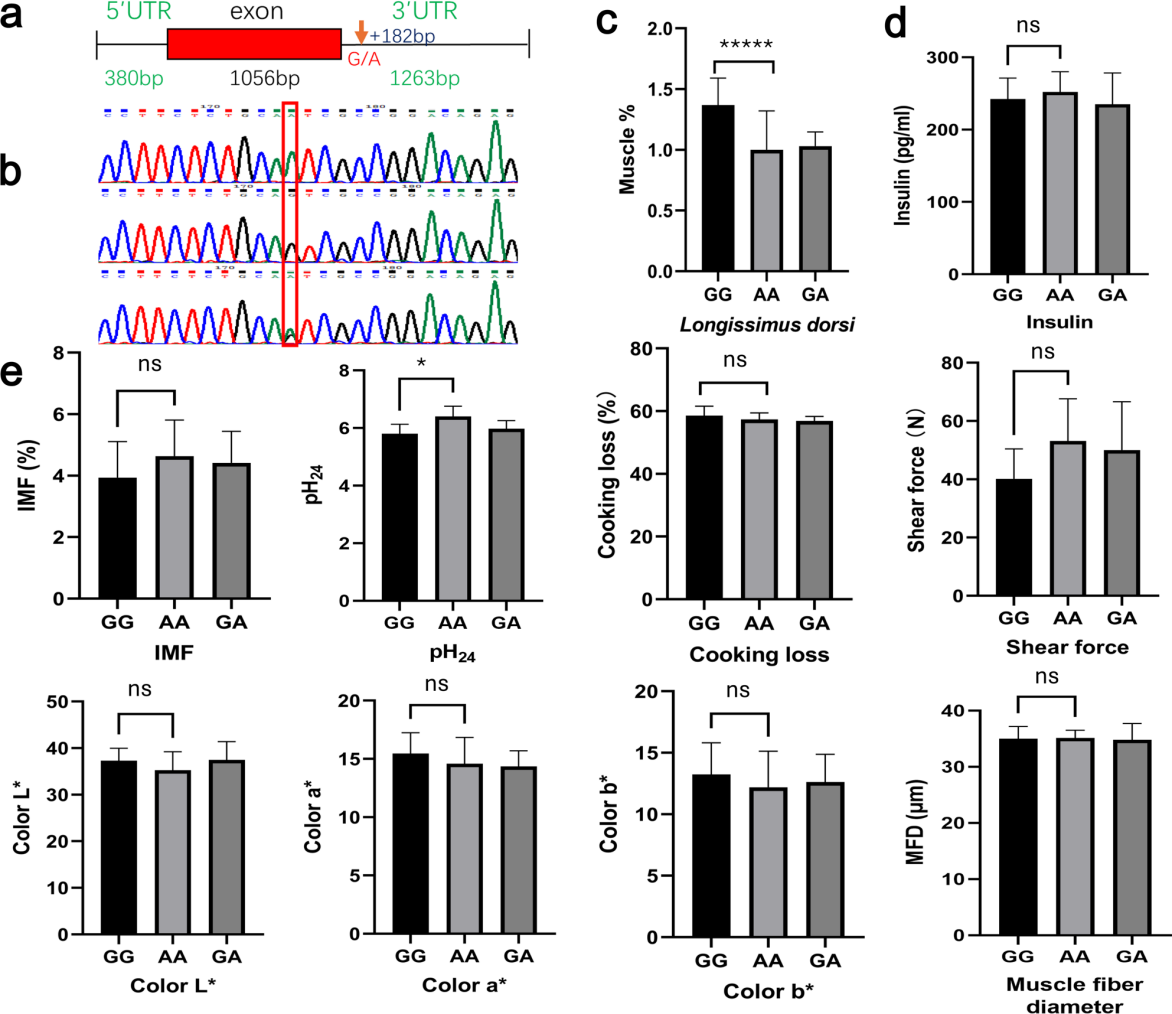
Transcriptome analysis of the *longissimus dorsi* muscle from homozygous AA and GG genotypes of the *MAFA* gene (n = 5). **a** Volcano plot of differentially expressed genes. **b** Principal component analysis (PCA) of gene expression profiles. **c** Heatmap of hierarchical clustering based on gene expression. **d–f** GO enrichment analysis of differentially expressed genes. **g** KEGG pathway enrichment analysis of differentially expressed genes (Top 10 pathways). **h** qRT-PCR validation of random selected differentially expressed genes (n = 3). Data are presented as mean ± SEM. Statistical significance is indicated as follows: ns, not significant; *P < 0.05; **P < 0.01; ***P < 0.005; ****P < 0.001; *****P < 0.0005

Using the GG genotype as the experimental group and AA as the control, DEGs were identified with adjusted *P* < 0.05 and a FC threshold of ≥ 2 or ≤ 0.5. A total of 966 DEGs were detected, with 959 genes upregulated and only 7 downregulated in the GG group (Fig. 3a). Principal component analysis (PCA) revealed distinct clustering between the two groups, indicating clear transcriptomic separation (Fig. 3b). Hierarchical clustering further highlighted distinct gene expression patterns between samples (Fig. 3c).

Gene Ontology (GO) enrichment analysis of the upregulated DEGs in GG individuals revealed significant enrichment in biological processes (BP) such as regulation of small GTPase-mediated signal transduction, histone modification, cell–matrix adhesion and assembly, and GTPase activity regulation (Fig. 3d). Molecular functions (MF) were enriched in protein serine/threonine kinase activity and GTPase regulator activity (Fig. 3e), while cellular components (CC) were enriched for cytoplasmic ribonucleoprotein granules, P-bodies, and transcription regulatory complexes (Fig. 3f). KEGG pathway enrichment analysis indicated that upregulated genes were significantly associated with cytoskeleton regulation, Ras signaling, and MAPK signaling pathways (Fig. 3g), all of which are closely associated with muscle growth and development, aligning with the observation that GG genotype individuals exhibit a significantly higher skeletal muscle proportion compared to AA individuals. Notably, small GTPase-mediated signal transduction and cytoskeletal regulation play central roles in muscle cell proliferation and differentiation, while the Ras and MAPK signaling pathways facilitate adaptive responses that promote muscle growth. These findings offer valuable insights into the molecular mechanisms underlying muscle development. Furthermore, the enrichment of P-body-related genes—key components in miRNA-mediated gene regulation—provides indirect evidence supporting the involvement of miRNA activity in the genotype-dependent phenotypic differences.

To validate the RNA-seq results, we randomly selected six DEGs for qRT-PCR validation. The expression trends observed were consistent with the RNA-seq data, confirming the reliability of the transcriptomic findings (Fig. 3h).

Given the small number of downregulated genes (n = 7) in the GG genotype, KEGG, and GO analyses for this group yielded limited enrichment in the CC category. Nevertheless, biological processes such as coenzyme A biosynthesis, phosphate ion homeostasis, and trivalent inorganic anion homeostasis were significantly enriched. In the molecular function category, sodium-phosphate symporter activity, neurexin family protein binding, and myristoyl-CoA hydrolase activity were significantly enriched.

The downregulation of coenzyme A biosynthesis may suggest a metabolic shift in muscle cells toward alternative energy sources, such as glycogen, potentially related to MAFA’s known role in insulin secretion. Reduced sodium-phosphate symporter activity may influence intracellular phosphate levels, affecting muscle cell proliferation and differentiation. Decreased neurexin family protein binding could reflect altered intercellular signaling, potentially contributing to muscle growth. Finally, suppression of myristoyl-CoA hydrolase activity may reduce fatty acid accumulation, thereby promoting muscle cell growth and function.

### The transcription factor MAFA targets the GHR gene to promote muscle growth

To elucidate the regulatory mechanism by which MAFA affects skeletal muscle mass, we first aimed to identify its direct target genes. Using previously obtained MAFA ChIP-seq data from ovine *longissimus dorsi* muscle [15] (SRA: PRJNA1135757), we employed MACS2 software for peak calling, setting a threshold of adjusted *P* < 0.05 and FC > 2 or < 0.5. Significant MAFA-binding peaks were mapped to the sheep genome (NC_056062.1), and 514 genes were annotated with MAFA binding sites within their promoter regions.

**Fig. 4:**
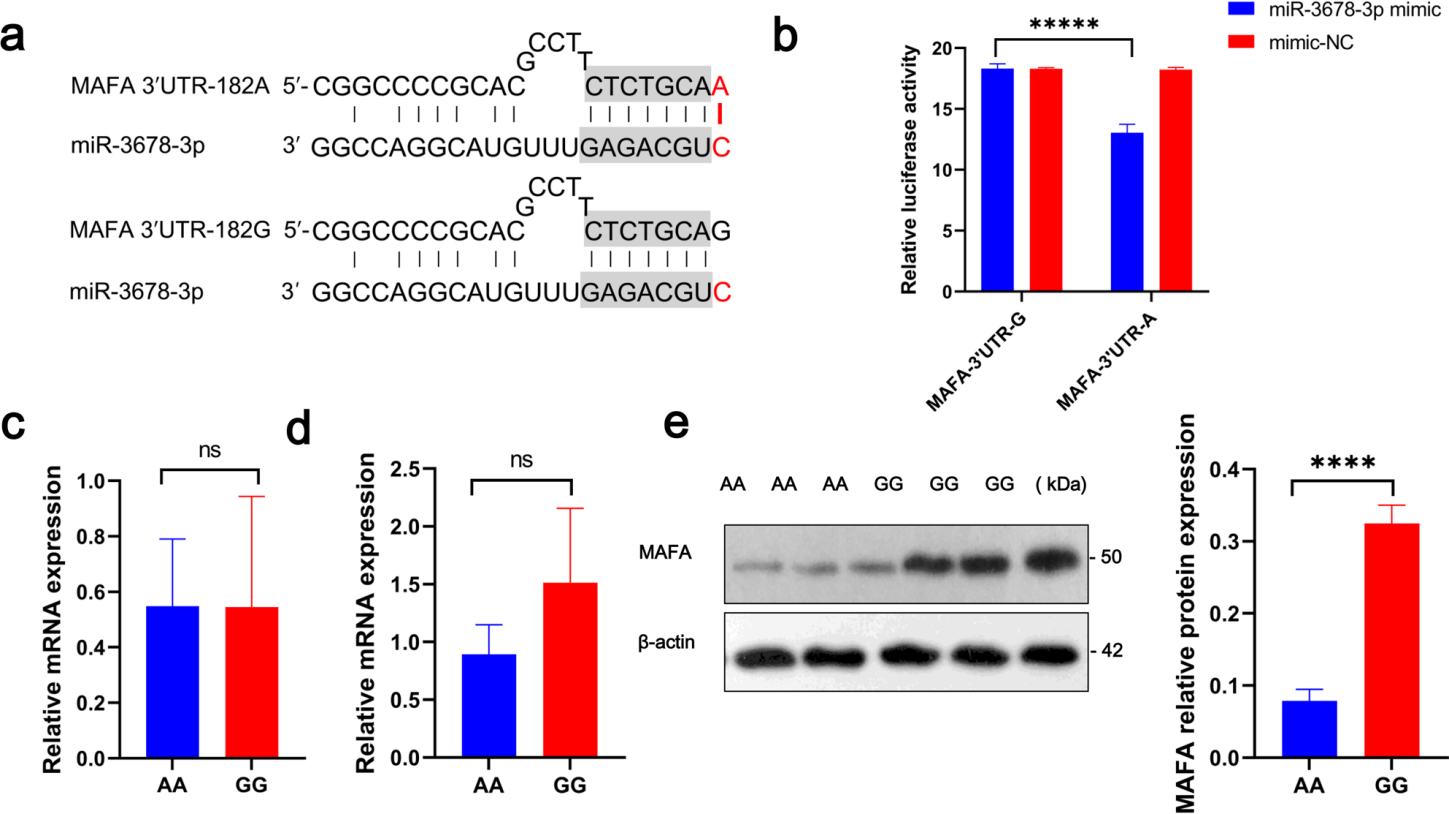
The transcription factor MAFA targets the *GHR* gene to promote muscle growth. **a** Venn diagram showing the overlap between MAFA target genes identified by ChIP-seq and differentially expressed genes from transcriptome analysis. **b** ChIP-seq peak profile indicating binding of MAFA to the *GHR* gene. **c** Heatmap showing Pearson correlation coefficients among expression levels of GHR and MAFA (FPKM values) and the proportion of *longissimus dorsi* muscle (n = 10). **d** Western blot analysis validating the chromatin immunoprecipitation (ChIP) efficiency of MAFA. Input: Cell lysate after centrifugation; SN: Supernatant after immunoprecipitation; IP: Antibody-magnetic bead complex after incubation and washing; IgG: IgG-magnetic bead complex after incubation and washing. **e** ChIP-PCR results showing significant enrichment of the *GHR* gene by MAFA (n = 3). **f**–**h** Western blot analysis of GHR expression and JAK2 phosphorylation levels in the *longissimus dorsi* muscle of individuals with different genotypes (n = 3, same batch of samples as in Fig. 2e, with shared β-actin internal control). Data are presented as mean ± SEM. Statistical significance is indicated as follows: ns, not significant; *P < 0.05; **P < 0.01; ***P < 0.005; ****P < 0.001; *****P < 0.0005

By intersecting these 514 candidate genes with the 966 DEGs identified from transcriptome comparisons between GG and AA genotypes, we identified 66 overlapping genes (Fig. 4a), including *GHR* and phosphoinositide-3-kinase regulatory subunit 1 (*PIK3R1*) (Table S3). Correlation analysis showed that *GHR* expression was positively correlated with both *MAFA* expression and the proportion of *longissimus dorsi* muscle (Fig. 4c). Given the established role of GHR in promoting growth, we hypothesized that *GHR* is a direct downstream effector of MAFA-mediated regulation of muscle development.

ChIP-seq results revealed two prominent MAFA binding peaks within the promoter region of *GHR* in the immunoprecipitation (IP) group compared to the control group without antibody treatment (input) (Fig. 4b), suggesting direct transcriptional regulation by MAFA. To validate this interaction, ChIP-PCR assays were performed. Western blot analysis showed a specific MAFA band at 50 kDa in the MAFA IP group compared to the IgG control (Fig. 4d), and PCR amplification demonstrated significantly higher enrichment of the *GHR* promoter in the MAFA group (Fig. 4e), confirming the specific binding of MAFA to the *GHR* promoter.

GHR, a member of the interferon-γ receptor superfamily, plays a key role in cellular signal transduction. Upon growth hormone (GH) binding at the cell surface, GHR activates JAK2 via phosphorylation, subsequently triggering downstream signaling pathways that modulate cellular growth and metabolism. Western blot analysis of *longissimus dorsi* muscle from individuals with different genotypes revealed significantly higher GHR protein levels in the GG group compared to the AA group (*P* < 0.001, Fig. 4f–g), along with elevated JAK2 phosphorylation levels (*P* < 0.005, Fig. 4h). These findings collectively support the hypothesis that MAFA promotes muscle growth by directly targeting the *GHR* gene and activating downstream JAK2 signaling, thereby contributing to the genotype-associated differences in muscle mass.

### Transfection of miR-3678-3p into myoblasts reduces MAFA and GHR expression

**Fig. 5:**
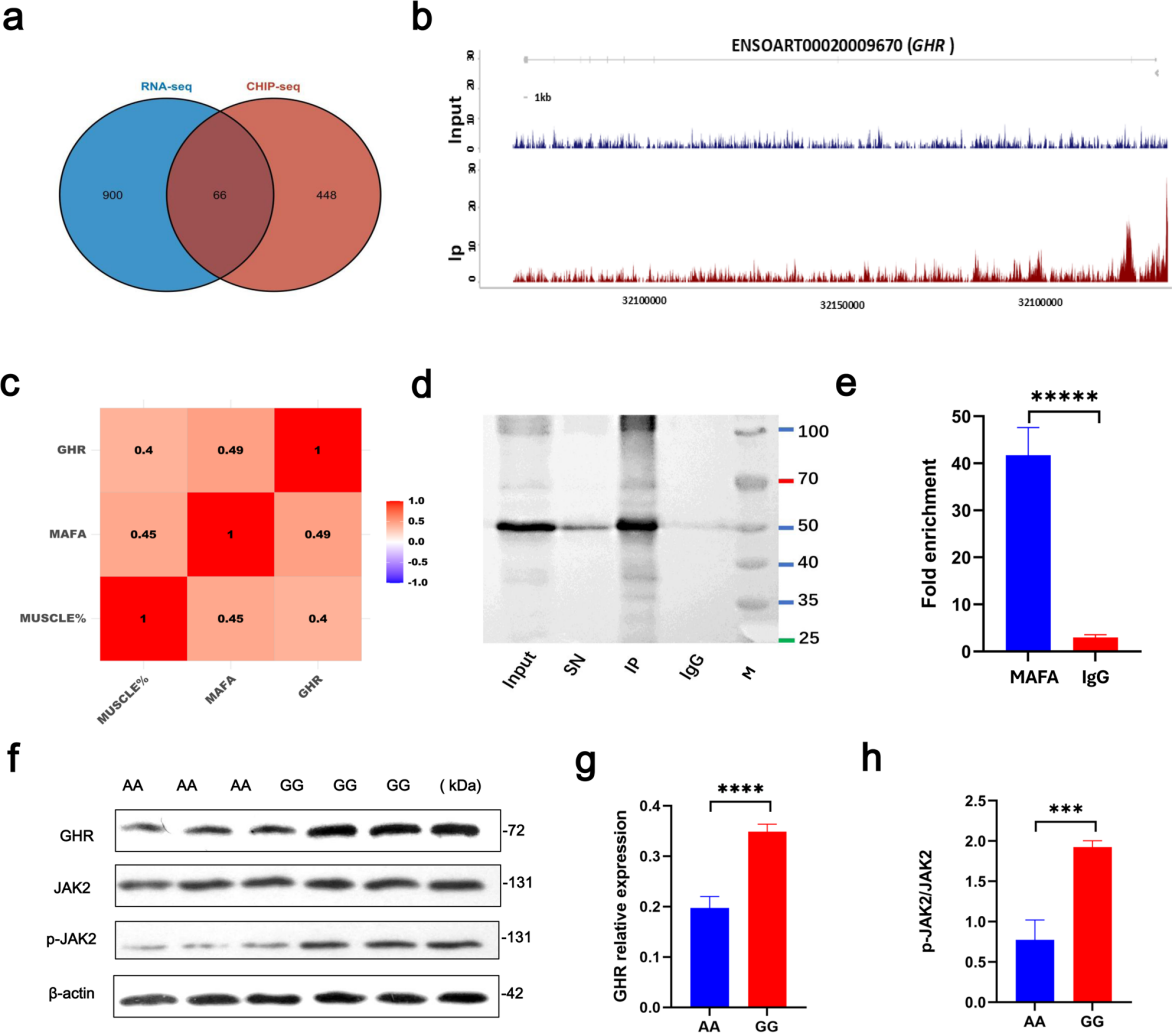
MiR-3678-3p transfection into myoblasts regulates *MAFA* expression and inhibits cell proliferation. **a** qRT-PCR analysis of *MAFA* mRNA expression. **b** Bar graph of MAFA protein levels from Western blot analysis. **c** qRT-PCR analysis of *GHR* mRNA expression. **d** Bar graph of GHR protein levels from Western blot analysis. **e** Bar graph showing the phosphorylation ratio of JAK2. **f** Western blot analysis of MAFA, GHR, JAK2 and phosphorylated JAK2 (p-JAK2). **g** EdU staining to assess cell proliferation. Proliferating cells were labeled with EdU (green), and all nuclei were stained with Hoechst 33,342 (blue). **h** Immunofluorescence staining to evaluate myogenic differentiation. **i** Flow cytometry analysis of cell cycle distribution following miR-3678-3p transfection. Bar graph data are presented as mean ± SEM (n = 3). Statistical significance is indicated as follows: ns, not significant; *P < 0.05; **P < 0.01; ***P < 0.005; ****P < 0.001; *****P < 0.0005

To further validate the regulatory mechanism whereby miR-3678-3p modulates *MAFA* translation and thus influences *GHR* expression, we isolated primary ovine myoblasts derived from individuals homozygous for the AA genotype at the g.1618 G/A locus. These homogeneous genotype cells were used for all subsequent miRNA transfection and *MAFA* overexpression assays to ensure a consistent genetic background. We transfected cells with a miR-3678-3p mimic or a negative control (NC) and performed downstream assays. Compared to the NC group, miR-3678-3p transfection did not significantly alter *MAFA* mRNA expression levels (Fig. 5a); however, Western blotting revealed a marked reduction in MAFA protein levels (*P* < 0.05, Fig. 5b and f), consistent with the anticipated translational repression of *MAFA* by miR-3678-3p.

Furthermore, both the mRNA (*P* < 0.05, Fig. 5c) and protein levels (*P* < 0.05, Fig. 5d and f) of *GHR* were significantly decreased following miR-3678-3p transfection. Concordantly, the phosphorylation level of JAK2 (p-JAK2/JAK2) was also significantly reduced (*P* < 0.001, Fig. 5e and f), supporting the regulatory pathway in which MAFA modulates *GHR* expression and downstream JAK signaling.

To assess cellular function, we performed EdU incorporation assays, which revealed a markedly lower proportion of EdU-positive cells in the miR-3678-3p group than in controls (*P* < 0.05, Fig. 5g). Immunofluorescence analysis showed that the differentiation and fusion indices of transfected cells were significantly higher than those of control cells (*P* < 0.01, Fig. 5h). Consistently, flow-cytometric analysis demonstrated a pronounced increase in the proportion of cells in the G1 phase (*P* < 0.05), accompanied by a marked decrease in S-phase cells (*P* < 0.05), with no significant change in the G2-phase population (Fig. 5i). Together, these findings indicate that miR-3678-3p suppresses myoblast proliferation while promoting differentiation owing to reduced MAFA protein levels.

### Effects of MAFA overexpression on myoblasts

**Fig. 6:**
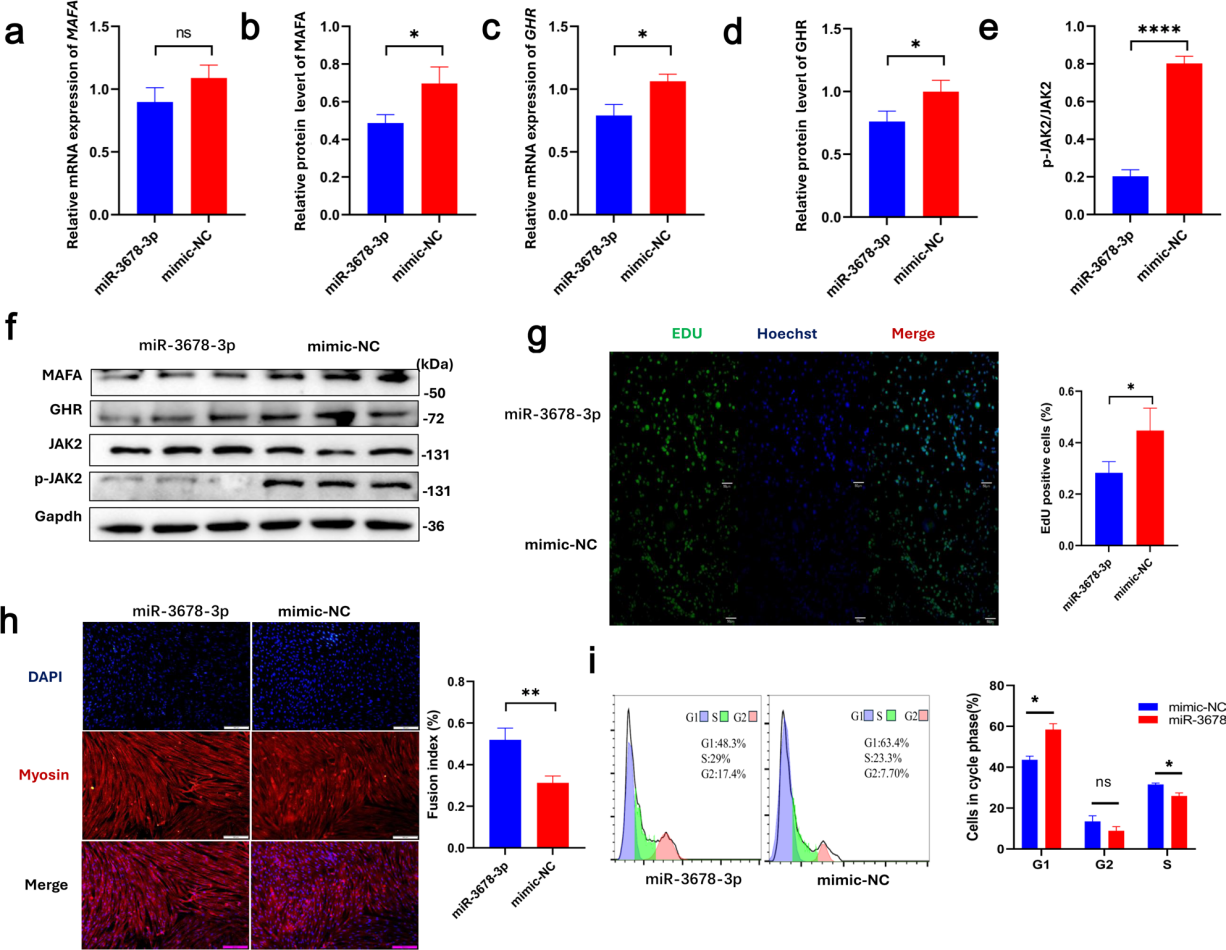
*MAFA* overexpression upregulates *GHR* expression and JAK2 phosphorylation, thereby promoting myoblast proliferation. **a** qRT-PCR analysis of *MAFA* mRNA expression. **b** Bar graph of MAFA protein levels from Western blot analysis. **c** qRT-PCR analysis of *GHR* mRNA expression. **d** Bar graph of GHR protein levels from Western blot analysis. **e** Bar graph showing the phosphorylation ratio of JAK2. **f** Western blot analysis of MAFA, GHR, JAK2, and phosphorylated JAK2 (p-JAK2). **g** EdU staining to evaluate cell proliferation. Proliferating cells were labeled with EdU (green), and all nuclei were stained with Hoechst 33,342 (blue). **h** Immunofluorescence staining to evaluate myogenic differentiation. **i** Flow cytometry analysis of cell cycle distribution following *MAFA* overexpression. Bar graph data are presented as mean ± SEM (n = 3). Statistical significance is indicated as follows: ns, not significant; *P < 0.05; **P < 0.01; ***P < 0.005; ****P < 0.001; *****P < 0.0005

To investigate the effects of MAFA on myoblast function, ovine myoblasts were transfected with either a *MAFA* overexpression plasmid or an empty vector as control. qPCR and Western blot analyses confirmed successful overexpression of *MAFA* at both the mRNA (*P* < 0.05, Fig. 6a) and protein levels (*P* < 0.05, Fig. 6b and f), with significantly higher expression observed in the overexpression group compared to the control.

We next examined the expression of *GHR*, a known downstream target of MAFA. Both *GHR* mRNA (*P* < 0.005, Fig. 6c) and protein levels (*P* < 0.05, Fig. 6d and f) were significantly increased in the MAFA-overexpressing group, further supporting the transcriptional regulatory role of MAFA on *GHR*. In addition, the phosphorylation level of JAK2, a key component of the downstream signaling cascade, was also significantly elevated following *MAFA* overexpression (*P* < 0.05, Fig. 6e and f).

Compared with the control group, EdU incorporation assays showed that *MAFA* overexpression led to a significant increase in cell proliferation (*P* < 0.05, Fig. 6g). Immunofluorescence analysis revealed that *MAFA* overexpression reduced the fusion indices of myoblasts (*P* < 0.05, Fig. 6h). Flow cytometry analysis further revealed that *MAFA* overexpression resulted in a significant decrease in the proportion of cells in the G1 phase (*P* < 0.005) and a corresponding increase in the S phase (*P* < 0.05), while no significant change was observed in the G2 phase (Fig. 6i). These findings indicate that MAFA inhibits myoblast differentiation while promoting myoblast proliferation by upregulating *GHR* expression and activating the JAK signaling pathway.

**Fig. 7:**
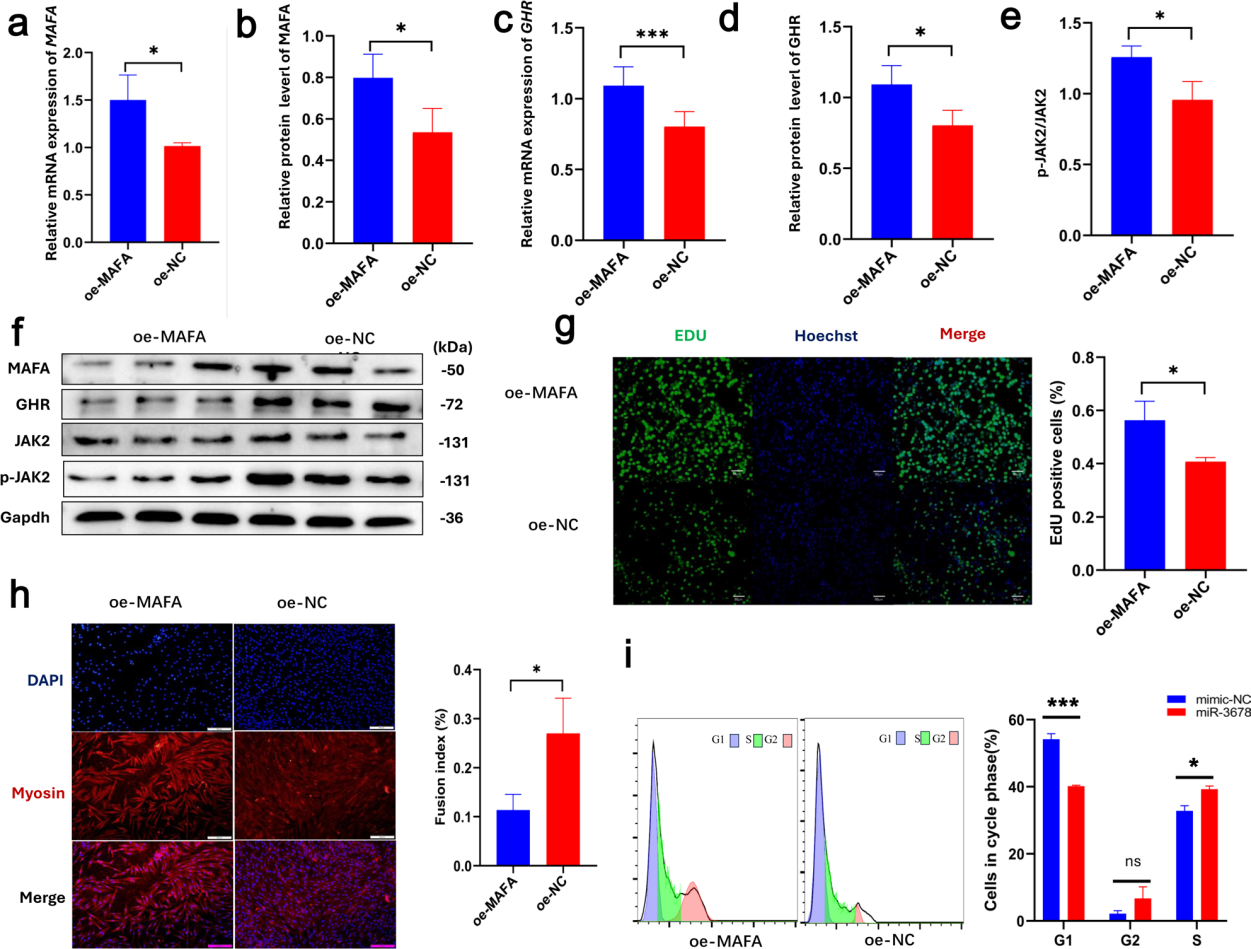
Schematic model illustrating the mechanism by which miR-3678-3p targets *MAFA* to regulate *GHR* expression and influence myoblast growth

Together, these findings delineate a coherent regulatory axis in which miR-3678-3p acts upstream to suppress *MAFA* translation, leading to reduced *GHR* expression and diminished JAK2 activation, thereby inhibiting myoblast proliferation and facilitating differentiation. Conversely, *MAFA* overexpression enhances *GHR* transcription and JAK2 phosphorylation, promoting proliferation while restraining early differentiation. This integrated miR-3678-3p-*MAFA-GHR* signaling cascade thus establishes a mechanistic framework underlying genotype-dependent regulation of muscle growth (Fig. 7).

## Discussion

Meat yield is a critical determinant of production efficiency in livestock. Although numerous major genes and molecular markers associated with animal growth traits have been identified, most are linked to general growth rate or body weight. In contrast, enhancing the proportion of skeletal muscle offers a more precise and practically valuable target for genetic selection [27, 28]. However, due to the lack of accurate in vivo measurement methods for skeletal muscle proportion, traditional carcass-based evaluations suffer from low selection intensity, ultimately limiting genetic progress. Therefore, the development of molecular markers for skeletal muscle proportion holds significant importance [29, 30].

Unfortunately, such markers remain scarce. While polymorphisms in the *MSTN* gene have been widely validated to directly affect muscle mass, favorable genotypes are often associated with lower intramuscular fat content, leading to inferior meat quality [31, 32]. In this study, we identified a polymorphism (g.1618 G > A) in the 3′ UTR of the *MAFA* gene that is significantly associated with the proportion of *longissimus dorsi* muscle in sheep. Notably, this polymorphism does not influence intramuscular fat content or other meat-quality traits, making *MAFA* a promising complementary candidate gene when the goal is improving muscle mass in sheep breeding programmes.

To elucidate the regulatory mechanism by which the g.1618 G/A polymorphism affects muscle growth, we predicted that this locus creates an 8-nucleotide seed pairing site (8-mer) for miR‑3678‑3p binding within the 3′ UTR of MAFA. In miRNA biology, nucleotides 2–7 of the miRNA constitute the seed region and pair with their targets through Watson–Crick base-pairing, while the first nucleotide at the 5′ end typically favours pairing with an adenosine, which is a conserved feature among vertebrates. Among the various seed-match types, 8-mer sites, defined by perfect pairing from positions 2–8 plus an adenosine opposite position 1, are considered the most specific and effective. In our case, the G allele at g.1618 aligns only with the miRNA positions 2–7, whereas the A allele introduces an adenosine opposite position 1 and thus generates a canonical 8-mer interaction with miR-3678-3p. Our dual-luciferase reporter assay confirmed that, although miR-3678-3p can bind both alleles, its binding to the A allele was significantly stronger — consistent with the observed greater translational repression of MAFA in the A allele context. Given that miRNA-mRNA interactions are dynamic and competitive, this allele-dependent difference in binding strength likely contributes to the phenotypic variation observed.

Post-transcriptional regulation via miRNA binding to the 3′ UTR of target genes is a key mechanism that typically leads to mRNA degradation or translational repression. To examine whether this mechanism underlies the differential expression of *MAFA* among genotypes, we measured *MAFA* expression in individuals carrying different alleles at the g.1618 locus. Quantitative PCR revealed no significant differences in mRNA levels between genotypes. However, Western blot analysis showed significantly higher MAFA protein levels in individuals with the GG genotype compared to those with the AA genotype, indicating that the genotype-dependent regulation of *MAFA* occurs at the post-transcriptional level. These findings confirm the upstream regulatory mechanism of the g.1618 G/A polymorphism in the *MAFA* 3′ UTR.

To explore the downstream consequences of this mutation, we integrated transcriptomic data from *longissimus dorsi* muscle tissues of animals with different genotypes and our previously generated MAFA ChIP-seq dataset [15]. Using ChIP-PCR validation, we identified *GHR* as a direct transcriptional target gene of MAFA. Both qPCR and Western blot results confirmed that *GHR* expression is significantly elevated in GG genotype individuals compared to AA genotype individuals. For subsequent functional experiments, primary myoblasts were isolated from individuals homozygous for the AA genotype, providing a consistent cellular genetic background to evaluate the mechanistic effects of miR-3678-3p and *MAFA*. These results were further supported by in vitro experiments, in which transfection with miR-3678-3p led to decreased *MAFA* and *GHR* expression, while *MAFA* overexpression increased *GHR* levels. Although our earlier study reported *EGFR* and *INSR* as MAFA-associated genes, the MAFA-binding sites for both were located within intronic regions—suggesting limited capacity for transcriptional regulation [15]. In contrast, leveraging ChIP-Seq data from our previous publication, together with ChIP-PCR validation in this study, we identified *GHR*—a gene well established to regulate animal growth—as a direct transcriptional target of MAFA, with MAFA binding to its promoter region.

*GHR* gene encodes a transmembrane receptor that binds GH and plays a central role in signal transduction. Upon GH binding, GHR undergoes conformational changes and forms dimers or oligomers, activating intrinsic tyrosine kinase activity [33–35]. Activated GHR directly stimulates JAK2, which phosphorylates STAT proteins, allowing them to act as nuclear transcription factors that promote cell growth and metabolism [36–38]. Phosphorylated GHR can also recruit SHC (Src homology 2 domain-containing) proteins, which are subsequently phosphorylated by JAK2 at specific tyrosine residues [39–41]. Activated SHC interacts with signaling molecules such as GRB2 (growth factor receptor-bound protein 2) to trigger Ras-GTPase activity and initiate the Ras-MAPK pathway, ultimately influencing cell proliferation and differentiation [42–44], consistent with our transcriptomic enrichment analyses. Moreover, JAK2 can phosphorylate IRS (insulin receptor substrate) proteins [45–47], thereby activating downstream signaling cascades including the PI3K/Akt pathway, which is critical for metabolic regulation [48, 49].

Given the central role of JAK2 phosphorylation in mediating GHR function, we examined JAK2 and phosphorylated JAK2 (p-JAK2) levels in *longissimus dorsi* muscle tissue of individuals with different *MAFA* genotypes. Western blot analysis revealed significantly higher levels of p-JAK2 in GG genotype individuals compared to those with the AA genotype. This trend was recapitulated in cellular assays. Primary myoblasts homozygous for the AA genotype, which exhibits high miR-3678-3p binding efficiency, were used as experimental materials. Cells transfected with miR-3678-3p exhibited reduced JAK2 phosphorylation and lower proliferation rates relative to controls, whereas *MAFA*-overexpressing cells displayed increased JAK2 phosphorylation and enhanced proliferation, yet concurrently suppresses first-phase differentiation. This dichotomy reflects the stage-dependent roles of JAK/STAT signaling in myogenesis. JAK2 activation initially favors expansion of progenitor cells, sustained or context-shifted signaling later triggers differentiation programs[50]. These findings demonstrate that MAFA, through genotype-dependent regulation and transcriptional activation of GHR, promotes JAK2-mediated signaling and myoblast proliferation (Fig. 7).

## Conclusions

In summary, the G > A polymorphism at position g.1618 in the 3′ UTR of the *MAFA* gene alters the binding affinity of miR-3678-3p, resulting in post-transcriptional regulation of MAFA protein expression. MAFA directly activates the transcription of its downstream target gene *GHR*, which in turn promotes JAK2 phosphorylation. This signaling cascade enhances myoblast proliferation and contributes to skeletal muscle growth.

## Supplementary Information

Below is the link to the electronic supplementary material.

Supplementary Material 1

Supplementary Material 2

Supplementary Material 3

## Data Availability

Primer sequences used for RT-qPCR experiments are provided in Table S1 The RNA-seq data have been submitted to the NCBI Sequence Read Archive (SRA) under BioProject accession number PRJNA1210790.
