# Supplementary material for: A 3′ UTR polymorphism g.1618 G > A in the MAFA gene modulates miR-3678-3p binding and enhances meat production in sheep via the MAFA/GHR/JAK2 pathway
Source: Genet Sel Evol. 2025 Dec 22;57:73. doi: 10.1186/s12711-025-01024-7 (PMC12751866; doi:10.1186/s12711-025-01024-7)
Supplement: Supplementary file 1 — Supplementary Material 1 [file 12711_2025_1024_MOESM1_ESM.pdf]

Table S1. Primer sequences used for RT-qPCR and ChIP-PCR

Target gene	Primer sequences (5′→3′)	Product size (bp)	Usage
miR-3678-3p	Forward: CGCGCTGCAGAGTTTGTACG	81	RT-qPCR
	Reverse: AGTGCAGGGTCCGAGGTATT		
MAFA	Forward: TGAACGATTTCGACCTGATG	76	RT-qPCR
	Reverse: GGCAGTCGGTGGCAAAAG		
MAFA	Forward: GCCCGACTTCTTCCTGTGA	301	DNA-PCR
	Reverse: CCGAGCAGAGGAGTTGGA		
PPARGC1A	Forward: CCTCAGTTCTGTCCGTGTTGTGTC	131	RT-qPCR
	Reverse: GCAGAGAGTATGAGAAGCGGGAATC		
IGFIR	Forward: GCGCAGGGGAGTCGTTC	120	RT-qPCR
	Reverse: CCATCCCGGAAGCAACTCTT		
NR3C1	Forward: TGGAATAGATGCCAGGGGTC	177	RT-qPCR
	Reverse: TAGGCAGAGTTTGGGAGGTG		
ACOT7	Forward: AGCAGGAGGGCTAGAAGATTT	106	RT-qPCR
	Reverse: AGGGGCCACTTCATCAGTCTA		
MCHR1	Forward: GAGAGCTGGATGGACCTTCG	81	RT-qPCR
	Reverse: TAGCAGGTGTAGGGCTCAGT		
C8orf37	Forward: GCAACATGGGGGCTCTTAGT	93	RT-qPCR
	Reverse: GGACCTTCTTGGTGGTCCAG		
GHR	Forward: CCCTCAGTGGCTAGGTTGTG	121	ChIP-PCR
	Reverse: GGGTGGTTCTGAGGGAAAGG		
GHR	Forward: GTGAAGCCACACCAGCTTTC	116	RT-qPCR
	Reverse: TTCAGGTGAACGGCACTTGG		
ACTB	Forward: TTCTAGGCGGACTGTTAG	84	RT-qPCR
	Reverse: TGCCAATCTCATCTCGTT		
