# Supplementary material for: A 3′ UTR polymorphism g.1618 G > A in the MAFA gene modulates miR-3678-3p binding and enhances meat production in sheep via the MAFA/GHR/JAK2 pathway
Source: Genet Sel Evol. 2025 Dec 22;57:73. doi: 10.1186/s12711-025-01024-7 (PMC12751866; doi:10.1186/s12711-025-01024-7)
Supplement: Supplementary file 2 — Supplementary Material 2 [file 12711_2025_1024_MOESM2_ESM.pdf]

Table S2. Analysis of variance and multiple comparison results for genotype-dependent traits

Trait	n	Model			Multiple comparison					
		P value	η²	95% CI	Comparison	Mean ± SEM (Group1)	Mean ± SEM (Group2)	P value	95% CI	Cohen's d
Longissimus dorsi (%)	230	6.31E-17	0.36	[0.25,0.46]	GG vs. AA	1.37 ± 0.03	1.00 ± 0.05	4.51E-22	[0.26,0.47]	2.04
					GG vs. GA	1.37 ± 0.03	1.03 ± 0.01	2.01E-18	[0.28,0.39]	1.39
					GA vs. AA	1.03 ± 0.01	1.00 ± 0.05	0.51	[-0.06,0.13]	0.16
Insulin (pg/ml)	60	0.39	0.03	[0.00,0.14]	GG vs. AA	242.38 ± 6.83	252.18 ± 8.03	0.36	[–31.54,11.94]	–0.34
					GG vs. GA	242.38 ± 6.83	235.23 ± 7.89	0.49	[–13.85,28.17]	0.19
					GA vs. AA	235.23 ± 7.89	252.18 ± 8.03	0.14	[–39.91,5.99]	–0.43
Shear force (N)	30	0.30	0.10	[0.00,0.29]	GG vs. AA	43.19 ± 5.53	52.23 ± 4.65	0.28	[–25.00,6.90]	–0.23
					GG vs. GA	43.19 ± 5.53	50.43 ± 8.26	0.53	[–29.70,15.20]	–0.23
					GA vs. AA	50.43 ± 8.26	52.23 ± 4.65	0.81	[–26.50,22.90]	–0.06
pH_{24}	30	0.01	0.33	[0.05,0.52]	GG vs. AA	5.85 ± 0.13	6.21 ± 0.13	0.02	[–0.65,0.07]	–0.30
					GG vs. GA	5.85 ± 0.13	6.12 ± 0.20	0.12	[–0.70,0.16]	–0.24
					GA vs. AA	6.12 ± 0.20	6.21 ± 0.13	0.60	[–0.62,0.44]	–0.07
Cooking loss (%)	30	0.20	0.11	[0.00,0.30]	GG vs. AA	58.82 ± 1.52	57.35 ± 0.83	0.49	[–3.80,6.80]	0.16
					GG vs. GA	58.82 ± 1.52	57.48 ± 1.95	0.63	[–5.50,8.20]	0.08
					GA vs. AA	57.48 ± 1.95	57.35 ± 0.83	0.94	[–6.30,6.60]	0.02
L*	30	0.47	0.06	[0.00,0.25]	GG vs. AA	37.25 ± 1.75	37.00 ± 1.14	0.91	[–4.10,4.60]	0.01
					GG vs. GA	37.25 ± 1.75	35.45 ± 3.17	0.61	[–6.7,10.3]	0.05
					GA vs. AA	35.45 ± 3.17	37.00 ± 1.14	0.68	[–10.0,7.00]	–0.06
a*	30	0.34	0.08	[0.00,0.27]	GG vs. AA	15.33 ± 1.04	14.48 ± 0.63	0.46	[–1.60,3.30]	0.09
					GG vs. GA	15.33 ± 1.04	13.92 ± 1.09	0.29	[–1.90,4.70]	0.13
					GA vs. AA	13.92 ± 1.09	14.48 ± 0.63	0.70	[–3.70,2.60]	–0.05
b*	30	0.71	0.03	[0.00,0.16]	GG vs. AA	12.48 ± 1.42	12.41 ± 0.86	0.97	[–3.10,3.30]	0.01
					GG vs. GA	12.48 ± 1.42	11.48 ± 1.28	0.61	[–3.70,5.70]	0.08
					GA vs. AA	11.48 ± 1.28	12.41 ± 0.86	0.68	[–4.60,2.80]	–0.09
IMF (%)	30	0.52	0.05	[0.00,0.23]	GG vs. AA	3.70 ± 0.40	4.70 ± 0.35	0.10	[–2.20,0.20]	–0.69
					GG vs. GA	3.70 ± 0.40	4.46 ± 0.74	0.44	[–2.30,0.80]	–0.37
					GA vs. AA	4.46 ± 0.74	4.70 ± 0.35	0.73	[–1.70,1.20]	–0.33
MFD (μm)	30	0.95	0.00	[0.00,0.05]	GG vs. AA	35.31 ± 0.78	34.63 ± 0.49	0.51	[–1.60,3.00]	0.12
					GG vs. GA	35.31 ± 0.78	34.98 ± 1.17	0.82	[–2.70,3.40]	0.03
					GA vs AA	34.98 ± 1.17	34.63 ± 0.49	0.83	[–2.60,3.30]	0.04
