# Supplementary material for: A 3′ UTR polymorphism g.1618 G > A in the MAFA gene modulates miR-3678-3p binding and enhances meat production in sheep via the MAFA/GHR/JAK2 pathway
Source: Genet Sel Evol. 2025 Dec 22;57:73. doi: 10.1186/s12711-025-01024-7 (PMC12751866; doi:10.1186/s12711-025-01024-7)
Supplement: Supplementary file 3 — Supplementary Material 3 [file 12711_2025_1024_MOESM3_ESM.pdf]

Supplementary Table S3. List of differentially expressed genes

Gene	FC	log_2FC	Adjusted P value	$log_{10}p$
PIK3R1	2.0181	1.0130	0.035633	1.4481
RSPRY1	2.0279	1.0200	0.015805	1.8012
MTDH	2.0312	1.0223	0.015293	1.8155
SOS2	2.0340	1.0243	0.009036	2.0440
SPEN	2.0588	1.0418	0.030428	1.5167
KMT2E	2.0722	1.0511	0.013159	1.8808
JMJD1C	2.1047	1.0736	0.016275	1.7885
DST	2.1047	1.0736	0.024845	1.6048
NUP58	2.1148	1.0805	0.018579	1.7310
TNRC6B	2.1161	1.0814	0.037237	1.4290
BCL9L	2.1574	1.1093	0.047629	1.3221
GHR	2.1817	1.1255	0.010837	1.9651
ZNF664	2.1856	1.1280	0.009028	2.0444
KDM2A	2.2016	1.1386	0.014990	1.8242
NEDD4	2.2189	1.1498	0.041516	1.3818
PLEKHA1	2.2224	1.1521	0.013659	1.8646
RBM24	2.2298	1.1569	0.027864	1.5550
PPFIBP2	2.2412	1.1643	0.030354	1.5178
KLF6	2.2593	1.1759	0.034629	1.4606
UBL3	2.2597	1.1762	0.023468	1.6295
PTPN21	2.2933	1.1974	0.044583	1.3508
KLF9	2.3006	1.2020	0.047911	1.3196
RPRD2	2.3101	1.2080	0.016281	1.7883
DAAM1	2.3161	1.2117	0.032299	1.4908
MBNL1	2.3227	1.2158	0.004438	2.3528
RNF111	2.3306	1.2207	0.012135	1.9159
PCMTD1	2.3744	1.2475	0.011163	1.9522
BAZ2B	2.4020	1.2642	0.023870	1.6221
MAPK6	2.4037	1.2652	0.015285	1.8157
RAVER2	2.4055	1.2664	0.010733	1.9693
BICRAL	2.5120	1.3288	0.015293	1.8155
ADAM19	2.5256	1.3366	0.047492	1.3234
FILIP1	2.5365	1.3429	0.016210	1.7902
ZFP36L2	2.5546	1.3531	0.019634	1.7070
PDP1	2.5693	1.3614	0.030775	1.5118
RPS6KA3	2.5795	1.3671	0.046286	1.3345
ANO6	2.5850	1.3701	0.017225	1.7638
TENT4B	2.6243	1.3919	0.015407	1.8123
LIMD1	2.6819	1.4232	0.041411	1.3829
SSH2	2.6951	1.4303	0.028295	1.5483
QKI	2.7121	1.4394	0.010422	1.9820

PHTF2	2.7768	1.4734	0.004898	2.3100
TNKS2	2.8280	1.4998	0.023870	1.6221
ZYG11B	2.8886	1.5304	0.016239	1.7894
TEAD1	2.9793	1.5750	0.013327	1.8753
RNF144B	2.9794	1.5750	0.015909	1.7984
PTPRD	2.9886	1.5795	0.021582	1.6659
AGL	3.1224	1.6427	0.038093	1.4192
CELF2	3.1334	1.6477	0.021666	1.6642
FNIP1	3.1645	1.6620	0.010733	1.9693
PPARGC1A	3.2724	1.7104	0.027182	1.5657
ATXN7	3.3000	1.7225	0.016239	1.7894
MED13	3.3691	1.7524	0.012595	1.8998
MAP3K20	3.3716	1.7534	0.007513	2.1242
XRN1	3.4029	1.7668	0.025305	1.5968
DDX6	3.4148	1.7718	0.013659	1.8646
MXD1	3.4778	1.7982	0.004438	2.3528
ASB15	3.5398	1.8237	0.018732	1.7274
PRKAB2	3.5710	1.8363	0.014946	1.8255
HIPK3	3.5779	1.8391	0.012370	1.9076
CDK19	3.6221	1.8568	0.012370	1.9076
ARRDC3	3.7338	1.9007	0.049317	1.3070
SOX6	3.8669	1.9512	0.016239	1.7894
NRIP1	3.9355	1.9765	0.024753	1.6064
AFF1	3.9711	1.9895	0.011942	1.9229
MFSD4B	5.2414	2.3900	0.011849	1.9263
